# Level of and Changes in Perceived Work Ability Among Partial Disability Pensioners and the Risk of Full Disability Pension—A Register-Linked Cohort Study

**DOI:** 10.1007/s10926-023-10161-z

**Published:** 2023-12-28

**Authors:** Mari-Anne Wallius, Tea Lallukka, Taina Leinonen, Jouko Remes, Jenni Ervasti

**Affiliations:** 1 Finnish Institute of Occupational Health, Helsinki, Finland; 2 Department of Public Health, University of Helsinki, Helsinki, Finland

**Keywords:** Work ability, Disability, Partial disability pension, Work ability score, WAS, Aging workers

## Abstract

**Purpose:**

To examine how the level of perceived work ability and its changes over time are associated with the risk of full disability pension (DP) among those receiving partial DP.

**Methods:**

We retrieved survey data on perceived work ability and covariates (sociodemographic factors and health behaviors) from a cohort study of Finnish public sector employees at two time points: 2008 and 2012 and linked them with register data on DP obtained from the Finnish Centre for Pensions up to the end of 2018. Participants had begun receiving partial DP in 2008 and responded to either the 2008 survey (n = 159) or both surveys (n = 80). We used Cox regression for the analyses.

**Results:**

During the follow-up, 61 (38%) of those receiving partial DP transitioned to full DP. Those with perceived poor work ability were at a higher risk of full DP (HR 1.93; 95% CI 1.11–3.38) than those with at least moderate work ability, after adjustment for covariates. During four years of receiving partial DP, perceived work ability decreased among 36% of the participants, and remained unchanged or improved among 64%. Change in work ability was not associated with a risk of full DP.

**Conclusion:**

Among those receiving partial DP, perceived poor work ability was a risk factor for full DP. Our findings highlight the importance of monitoring the level of perceived work ability of those receiving partial DP to enable identifying individuals at an increased risk of full DP.

**Supplementary Information:**

The online version contains supplementary material available at 10.1007/s10926-023-10161-z.

## Introduction

The share of older people in the population is continuing to increase in Europe [1]. Measures are needed to ensure that people are able to continue working until retirement age. Early retirement due to disability is a major cause of lost working years [2]. Helping people with reduced work ability to participate in work life is essential.

To be able to continue to work, an employee must have sufficient work ability. When full-time labor market participation is no longer possible, partial disability pension (DP) (granted on either a temporary or permanent basis) is one solution. Partial DP is granted temporarily on the premise that the employee will regain full work ability. The decision is permanent if this is unlikely [3].

Partial DP enables an employee to combine part-time work with partial absence from work. In Finland, partial DPs account for almost one third of all granted DPs [4]. The use of partial DP has increased in recent decades [5, 6], especially among public sector employees [7]. In 2021, 70% of those who began partial DP were at least 55 years old and 69% were women [4]. To receive partial DP, a person’s work ability must be reduced by at least 40% for at least one year due to a medically confirmed illness, injury, or impairment [3]. These numerical working capacity requirements [8] and partial disability practices vary across countries [9, 10]. However, in Finland, partial DP is related to long-term disability, and is also used as a longer-term solution [3], whereas partial sickness benefit, for instance, is granted in the earlier stage of illness and for a shorter period of time [11].

If the work ability of those receiving partial DP deteriorates to the extent that it is reduced by at least 60%, full DP may be granted [3]. Little evidence exists on the factors that predict transitioning from partial DP to full DP. A previous Finnish study examined labor market transitions among those receiving partial DP in the public sector and found that 10% transitioned to full DP each year and that only 2% returned to full-time work during a six-year follow-up [12]. A recent Finnish register study showed that male gender, older age and low education level were major risk factors for transitioning from partial to full DP [13].

Those receiving partial DP commonly continue working as well as receiving a pension [14]. To identify those at risk of deteriorating work ability, it is important to monitor work ability. The Work Ability Index (WAI) is an instrument used today in occupational health services and for research purposes worldwide to assess work ability [15]. Work ability is often conceptualized as the balance between work demands and individual resources [15, 16]. It can be measured using the first dimension of the WAI, that is, the Work Ability Score (WAS) which is a person’s self-assessment of their current overall level of work ability compared with lifetime best [16–18]. Poor work ability or a strong decline in WAS is a predictor of DP among aging municipal workers [19].

WAS-based work ability seems to decline with age [16, 20, 21] and health problems [22], but chronic diseases do not necessarily mean limited work ability [16]. Heterogeneity has also been reported in the association between work ability and retirement pathways [23]. The individual resources of those receiving partial DP have decreased due to health reasons and therefore the demands of their work have been eased, i.e., their working hours have been reduced to match their work ability. The average WAS-based work ability of those receiving partial DP was lower than that of the non-pensioner group, and the average level of work ability was also slightly lower while receiving partial DP than during the preretirement period [12]. However, to the best of our knowledge, how perceptions of work ability change over time while receiving partial DP, and how the level of and changes in perceived work ability are associated with the risk of full DP, remain unknown.

To help people continue to work with their remaining work ability until older age, we need to better understand work ability and its changes among those receiving partial DP. Our aim was to examine whether the risk of full DP varies according to the level of perceived work ability after a person has transitioned to partial DP and how changes in perceived work ability over time are associated with the risk of full DP.

## Methods

### Participants and Design

The data for this study were derived from the Finnish Public Sector (FPS) study, which is an ongoing prospective cohort study of employees in the municipal services of ten towns and six hospital and healthcare organizations [24, 25].

**Fig. 1:**
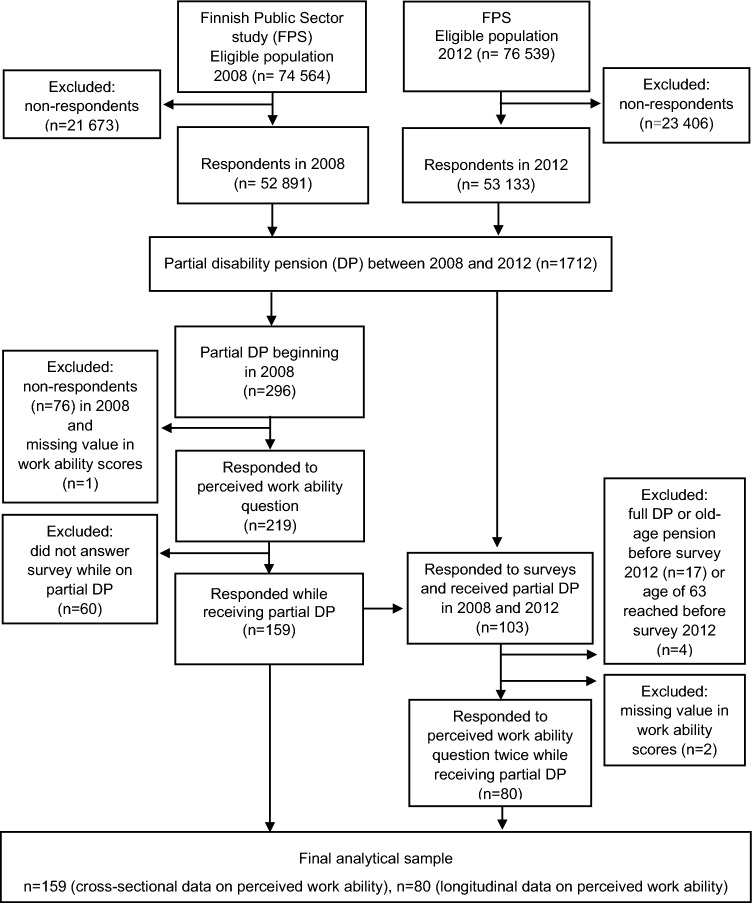
Flow chart of study participants

We used data obtained from the surveys collected in 2008 and 2012, which had an average response rate of 70%. The data on DP were obtained from the registers of the Finnish Centre for Pensions and linked prospectively (from 2008 until the end of 2018) to the survey data of the participants. For the purposes of this study, we focused on those who started receiving partial DP in 2008. We included participants who had data on perceived work ability in 2008 and who responded to the survey while receiving partial DP in our analyses. Participants with data on perceived work ability for both 2008 and 2012 were included in further analyses, providing that they had not transitioned to old-age pension or full DP, or reached the age of 63 before the 2012 survey. This resulted in analytical samples of 159 and 80 participants, respectively, as described in Fig. 1.

#### Ethical Consideration

Ethical approval was obtained from the Ethics Committee of the Helsinki and Uusimaa hospital district (HUS/1210/2016). The study was conducted in accordance with the Helsinki declaration. Informed consent was obtained from all the individual participants of the study.

#### Work Ability Score

Work ability was assessed using the WAS, in which the respondent assesses their current work ability compared to their lifetime best on a ten-point scale (0 = completely unable to work, 10 = work ability at its best) [16]. This first WAI item has shown to be strongly associated with overall WAI [18, 26] and can be considered a reasonable alternative to the seven-item WAI for evaluating work ability level and how it changes over time [18]. WAS is considered a reliable, valid, and responsive instrument for assessing work ability. The test-retest reliability intraclass correlation coefficient (ICC) has been 0.89 [27] and 0.83 [28]. The standard error of the measurement of 0.69 and the smallest detectable change of 1.92 points in sick-listed workers has been reported [27] as showing good agreement of the WAS. Acceptable responsiveness (e.g., sensitivity of 41.9% and specificity of 85.0% for WAS at cut-off point 5) has also been reported [19]. An association between a one-point decrease in WAS and a 33% higher risk of early retirement has been observed in the general working population [29].

To examine the association between the level of perceived work ability and the risk of full DP, we used WAS as a categorized variable (main analysis) and as a continuous variable (sensitivity analysis). We categorized the WAS scores for 2008 as follows: 0–5 points = poor work ability [16, 19, 21] and 6–10 points = at least moderate work ability.

Changes in perceived work ability were calculated as the four-year change in WAS by subtracting the 2008 score from the 2012 score. For descriptive purposes, we categorized the differences into three classes: (i) no change in work ability (work ability remained constant between the two timepoints), (ii) improved work ability (WAS increased by ≥ 1 point), and (iii) decreased work ability (WAS decreased by ≥ 1 point). Due to the small size of the sample, change in work ability was also dichotomized into unchanged or improved work ability and decreased work ability for tabulation.

#### Covariates

We obtained the covariates from the 2008 survey. They included age in years, gender, occupational class, marital status, and health behaviors such as smoking, leisure-time physical activity, alcohol consumption, and body mass index (BMI), as these have been associated with a higher risk of DP [29–31].

The occupations were classified according to the International Standard of Occupations codes [32] and categorized into three groups: (i) high (upper-grade nonmanual workers, e.g., managers), (ii) intermediate (lower-grade nonmanual workers, e.g., people working in customer services), and (iii) low (manual workers, e.g., cleaners). Marital status was dichotomized into “married/cohabiting” and “other” (i.e., unmarried, widow/widower or divorced) [33, 34]. To calculate BMI (weight in kilograms divided by height in meters squared), we used self-reported weight and height. Smoking was categorized into “non-smokers” or “current smokers” (at the time of the survey) [35]. Alcohol consumption was elicited by asking about weekly consumption, and alcohol intake was calculated as grams per week. Information on the participants’ average weekly hours spent on leisure-time physical activity during the last 12 months was elicited by four questions, from which we calculated the metabolic equivalent index (in MET hours a day).

#### Disability Pension

The register, maintained by the Finnish Centre for Pensions, contains all the pension recipients on the earnings-related pension scheme, which is the primary pension scheme in Finland. DP can be granted on either a temporary or permanent basis to a person aged 18–62 with a work history that has accrued a pension [3]. In this study, temporary and permanent DPs were pooled for the analyses [12], as return to work is relatively uncommon, even after temporary DP [36]. We focused on all-cause DP.

To analyze the level of work ability and the associated risk of full DP, the follow-up started on the day the 2008 survey was returned and continued until the end of 2018. Receipt of full DP was coded as an event (= 1). Cases were censored at the end of the follow-up on 12/31/2018, or when participants reached old-age pension or the age of 63 (when DP can no longer be granted), or died. To analyze the change in work ability over four years and the associated risk of full DP, the follow-up started on the day on which the Timepoint 2 survey was returned and ended on 12/31/2018, or when participants reached old-age pension, or the age of 63 years, or died.

#### Statistical Methods

We used Cox proportional hazards regression analysis to estimate hazard ratios (HR) with confidence intervals (95% CI) for full DP due to any cause. The proportional hazards assumption was evaluated graphically from Kaplan–Meier curves and deemed appropriate. Four models were constructed. In the case of a categorical variable, the first category was used as a reference. In Model 1, we analyzed the HRs and 95% CIs of the full DP connected with work ability level, adjusting for age and gender. Model 2 was adjusted for the same covariates as in Model 1 plus marital status and occupational class; Model 3 was adjusted for only smoking, alcohol consumption, and leisure-time physical activity; and Model 4 was adjusted for all the preceding covariates plus BMI. The models were estimated with WAS as a categorical variable in the main analyses and as a continuous variable in the sensitivity analyses. Two of the covariates, alcohol consumption and leisure-time physical activity, were logarithmically transformed. The proportion of missing values was below 2% for most covariates, except for BMI (4%) and smoking (3%). We conducted multiple imputation (n = 5) for the missing covariate data.

We used Cox regression to examine whether the changes in work ability between Timepoint 1 (2008) and 2 (2012) were associated with the risk of full DP over an average 3.5-year follow-up. Change was operationalized as the difference in WAS between Timepoints 1 and 2 (WAS_T2_ – WAS_T1_): positive values indicated improved WAS and negative values poorer WAS. Follow-up time was included as a log-transformed offset variable. Statistical analyses were performed using IBM SPSS version 27.0 (SPSS Inc., Chicago, Illinois, USA) and SAS version 9.4 (SAS Institute Inc., Cary, NC, USA).

## Results

Of the 296 individuals who transitioned to partial DP in 2008 (see Fig. 1), 159 (54%) responded to the survey in 2008 while on partial DP, and 61 (38%) of these ended up on full DP during follow-up. Table 1 presents the descriptive characteristics of the study sample. The mean age of the participants was 55.8 (SD 4.5) and 86% were women. At baseline, 55% scored five points or less on the WAS, 45% scored six points or more. Those with poor work ability made up 69% of the full DP events.

**Table 1:** Description of study variables by perceived work ability level groups (baseline 2008)

	All N* (%)	Perceived work ability level^a^	
		At least moderate work ability N* (%)	Poor work ability N* (%)
N	159	72	87
Women (%)	137 (86)	64 (89)	73 (84)
Age (mean)	55.8 (SD 4.5)	55.7 (4.9)	55.8 (SD 4.3)
Occupational class (%)			
High	66 (42)	37 (51)	29 (34)
Intermediate	48 (30)	23 (32)	25 (29)
Low	44 (28)	12 (17)	32 (37)
Marital status (%)			
Married/cohabiting	111 (70)	46 (65)	65 (75)
Other	47 (30)	25 (35)	22 (25)
Smoking (%)			
Non-smoker	126 (82)	57 (84)	69 (80)
Smoker (current)	28 (18)	11 (16)	17 (20)
Alcohol consumption^b, #^ (mean)	71.4 (SD 107.7)	65.0 (SD 95.7)	94.5 (SD 98.0)
Body mass index^c^ (mean)	27.2 (SD 4.7)	27.4 (SD 5.2)	27.0 (SD 4.3)
Physical activity^d, #^ (mean)	2.4 (SD 2.0)	2.7 (SD 2.1)	2.2 (SD 1.9)
Perceived work ability (mean)	5.2 (SD 1.9)	6.9 (SD 0.9)	3.8 (1.3)

*Numbers vary because some survey responses were missing

#Non-logarithmic transformed data presented

^a^Perceived work ability was assessed using the Work ability score (WAS), and categorized as 0–5 points = poor work ability and 6–10 points = at least moderate work ability

^b^Alcohol consumption calculated as grams per week

^c^Body mass index(BMI) calculated from self-reported weight and height

^d^Leisure-time physical activity, metabolic equivalent (MET) hours a day

Among the participants receiving partial DP, those with poor work ability were at a higher risk of full DP (HR 1.93; 95% CI 1.11–3.38), after adjustment for sociodemographic factors, health behaviors, and BMI (Table 2). Using the continuous exposure variable, we found that a one-point increase in perceived work ability was associated with a lowered risk of full DP of 27% (95% CI 63–85%) (Supplementary Table 1).

**Table 2:** Association between level of perceived work ability and risk of full disability pension (2008–2018) among those receiving partial disability pension (N = 159, N of events = 61)

	Level of perceived work ability*		
	At least moderate work ability (N=72)	Poor work ability (N=87)	
N of events	19	42	
		HR	95% CI
Model 1^a^	Ref.	2.02	1.17–3.48
Model 2^b^	Ref.	1.92	1.10–3.34
Model 3^c^	Ref.	2.04	1.19–3.51
Model 4^d^	Ref.	1.93	1.11–3.38

Hazard ratios (HR) and their 95% confidence intervals (CI)

*The level of perceived work ability was assessed using the Work ability score (WAS), and categorized as 0–5 points = poor work ability and 6–10 points = at least moderate work ability

^a^Level of perceived work ability, adjusted for baseline age and gender

^b^Level of perceived work ability, adjusted for sociodemographic factors (baseline age, gender, occupational class, and marital status)

^c^Level of perceived work ability, adjusted for health behaviors (baseline smoking, alcohol consumption and leisure-time physical activity)

^d^Level of perceived work ability, adjusted for baseline age, gender, occupational class, marital status, smoking, alcohol consumption, leisure-time physical activity, and body mass index (BMI). Alcohol consumption and leisure-time physical activity were logarithmically transformed. Leisure-time physical activity, BMI, marital status, occupational class, and smoking with imputed values

Over the four years between the study timepoints, work ability decreased among 36% of the study participants receiving partial DP, remained unchanged among 28% and improved among 36%. The mean change in WAS among those whose work ability decreased (n = 29), was − 2.1 points (SD 1.6) and the WAS score of the majority (79%) of these participants decreased by either one or two points. Correspondingly, the 43% WAS score of those whose work ability improved or remained unchanged (n = 51) increased by one or two points (mean change 1.2, SD 1.3). During the follow-up, 17 (21%) participants were granted a full DP, the work ability of 7 (24.1%) participants decreased, and the work ability of 10 (19.6%) participants remained unchanged or improved (see descriptive characteristics in Supplementary Table 2). The mean time for full DP was 3.6 (SD 1.32) years.

Regarding the change in work ability over four years, as the number of full DP events in the data was limited, we were unable to calculate the HR estimates for all the potential covariates with our model. As the number of men was small, we did not adjust the model for gender. We observed no association between change in work ability and the risk of full DP (HR 1.12; 95% CI 0.90–1.40) (Table 3).

**Table 3:** Hazard ratios (HR) and their 95% confidence intervals (CI) for associations between changes in perceived work ability from Timepoint 1 (2008) to Timepoint 2 (2012) and subsequent disability pension until the end of 2018. (N = 80, N of events = 17)

	Change in work ability* HR	95% CI
Model 1^a^	0.95	0.78–1.15
Model 2^b^	1.01	0.86–1.19
Model 3^c^	1.05	0.88–1.27
Model 4^d^	1.12	0.90–1.40

*The change in work ability score (WAS) over four years was assessed by subtracting the score in 2008 from the score in 2012. Positive values indicated improved WAS and negative values poorer WAS. Follow-up time was included as a log-transformed offset variable in all the models

^a^Change in work ability, adjusted for baseline age. As number of men was small, the model was not adjusted for gender

^b^Change in work ability, adjusted for sociodemographic factors (baseline occupational class and marital status). Model was not adjusted for age

^c^Change in work ability, adjusted for health behaviors (baseline smoking, alcohol consumption and leisure-time physical activity)

^d^Change in work ability, adjusted for baseline occupational class, marital status, smoking, alcohol consumption, leisure-time physical activity, and body mass index (BMI). The model was not adjusted for age. Alcohol consumption and leisure-time physical activity were logarithmically transformed. Leisure-time physical activity, BMI, marital status, occupational class, and smoking with imputed values

## Discussion

Using register data on DP events in 2008 − 2018 and survey data from 2008, we found that WAS-based work ability was associated with full DP over a maximum eleven-year follow-up among Finnish public sector employees who had begun receiving partial DP in 2008. The risk of full DP among those with poor work ability was 1.9 times that of those with at least moderate work ability. Our findings indicate that the association between low WAS-based work ability and register-based DP found in earlier studies of Finnish, mainly full-time employees [19, 21] also apply to those who have transitioned to partial DP.

Of our study participants receiving partial DP, who had an average age of 56, 55% scored five points or less on the WAS. In a previous study of general municipal employees aged 44–58 (N = 5251), the proportion of participants with poor WAS-based work ability was much lower––only 14% [19]. Respectively, in a study of Finnish employees in all sectors and occupations (N = 11 124) only 3% of the participants rated their work ability as poor [21]. The participants were younger than those in our study, which may explain the difference. As poor work ability is often related to chronic health problems among older employees [22], we expected the work ability ratings of those receiving partial DP among our study sample to be low. Impaired work ability may be explained by reduced health status, as receiving partial DP requires a 40% reduction in work ability due to a medically confirmed illness.

According to an earlier study, the average WAS of Finnish municipal employees was poor among those who transitioned to partial DP [12]. Our study deepened the understanding of work ability after beginning to receive partial DP and its changes over a period of four years. Work ability remained unchanged or even improved among 64% of those who continued on partial DP for this period. Although work ability is expected to decline with age, especially from midlife onwards [15, 20, 21], this declining trend is less pronounced when the WAI is low [23]. The work ability of our participants was often already poor or moderate at the onset of partial DP. We speculate that employees with already low scores may find it difficult to change their assessment of their work ability, and naturally, the score cannot decrease indefinitely. However, we found no significant floor or ceiling effects in our study. An earlier study of sick-listed workers with chronic pain, defined the ceiling or floor effect as more than 15% of individuals in a sample who scored the maximum or minimum WAS [27].

The personal resources of those receiving partial DP had decreased for health reasons, and their work demands and individual resources had been balanced by reduced working hours. This may be one of the reasons why the majority of the participants felt that their work ability remained constant or improved. However, there is also evidence that work ability remains stable over the years for the majority of older workers: A recent study showed that long-term WAS-based work ability from midlife onwards remained rather stable among the majority of employees over 16 years of follow-up [34]. Another study reported three trajectories of WAI-based work ability among older workers, and that the work ability of only 17% of them had declined over an approximately 12-year follow-up [23]. However, trajectory analysis also found nonlinear changes in WAI-based work ability from midlife onwards in a 28-year follow-up of municipal employees [37]. With only two measurement points of work ability, we were unable to identify such changes.

Although previous studies have indicated that employees with declining work ability tend to retire earlier [19, 20, 23], we found no evidence that a change in perceived work ability over four years was associated with the risk of full DP among employees receiving partial DP. This may partly be explained by the fact that the differences between the level of the groups’ work ability were small, the mean values at baseline being 5 (poor) and 6 (moderate). Further, the changes in both the increased and decreased WAS were minor, that is, an average change of 1 or 2 points per direction. A one-point decrease in WAS-based work ability has been associated with a 33% increased risk of early retirement [29]. However, direct comparison to our study is difficult due to the differences between the study samples (healthy versus those receiving partial DP).

### Strengths and Limitations of the Study

A major strength of the study was that the retirement data were derived from a complete national register, which makes our measure of DP valid and reliable. Second, we employed the well-validated and widely used WAS to ascertain the level of and change in perceived work ability during follow-up. The perceived physical and mental demands at work of those receiving partial DP need to be further investigated. In addition to individual characteristics, physical and psychosocial working conditions are known to partly explain the link between poor work ability and an increased risk of DP [38]. Although psychosocial factors are also related to a risk of DP [21], earlier studies have demonstrated that they are rather similar within occupations, as implied by the use of psychosocial job exposure matrixes [39, 40]. Thus, occupational status can be seen as a conservative proxy for psychosocial stressors, and we did not adjust models with psychosocial factors to avoid the potential overadjustment.

The study also has limitations. The sample sizes were small, as the groups were highly selective. Those who did not answer the survey’s work ability question twice or were not receiving partial DP while participating in the survey dropped out of the study sample. Indeed, only 80 participants gave two survey responses, and there were only 17 DP events. Ten outcome events per predictor variable (EPV) has been the rule of thumb as regards the validity of the regression models [41]. However, less than 10 EPV has also been suggested as acceptable [42]. Consequently, a Type II error might have occurred: This would mean an incorrect lack of association between a variable and outcome event due to low statistical power. Lack of statistical power may also explain why we found no evidence that a four-year change in work ability was associated with a risk of full DP. Therefore, these results should be interpreted with caution. In addition, the rather long interval between the two measurement points may have resulted in selection bias (e.g., survivorship bias) in our sample, that is, those with declined work ability may have transitioned to full DP during the four years of follow-up. Our results may underestimate the risk of full DP among those receiving partial DP whose work ability is declining.

It can be debated whether the single self-reported item we used to assess work ability captures all the physical and mental capacities that are essential factors for work ability. However, WAS is considered a valid instrument for measuring work ability [27, 28] and a reasonable alternative to the WAI for describing the risk of full DP [19]. It should also be noted that most (86%) of our study participants were women. In Finland, 69% of those receiving partial DP are women [4] and women constitute 80% of public sector employees [43]. Another limitation of this study is that these findings cannot really be generalized to employees in the private sector, in which men are largely represented. In addition, our results might not be directly generalizable to other countries that have different pension systems to those in Finland. However, partial DP might to some extent resemble prolonged partial sickness absence or similar rehabilitation benefits in other countries if the benefit requires a long-term health illness and allows participation in part-time work.

## Conclusion

Poor work ability was associated with a higher risk of full DP in a group that had recently transitioned to partial DP. Our findings underscore the importance of monitoring the level of the perceived work ability of those receiving partial DP, for instance by occupational health care, to facilitate identifying individuals at an increased risk of full DP and who may need special support to be able to continue at work.

### Supplementary Information

Below is the link to the electronic supplementary material.

Supplementary material 1 (DOCX 16.3 kb)

Supplementary material 2 (DOCX 17.2 kb)

## Data Availability

The datasets generated and analyzed during the present study are not publicly available.
