# Supplementary material for: Level of and Changes in Perceived Work Ability Among Partial Disability Pensioners and the Risk of Full Disability Pension—A Register-Linked Cohort Study
Source: J Occup Rehabil. 2023 Dec 28;34(3):707–15. doi: 10.1007/s10926-023-10161-z (PMC11364573; doi:10.1007/s10926-023-10161-z)
Supplement: Supplementary file 1 — Supplementary material 1 (DOCX 16.3 kb) [file 10926_2023_10161_MOESM1_ESM.docx]

Level of and changes in perceived work ability among partial disability pensioners and the risk of full disability pension––A register-linked cohort study

Journal of Occupational Rehabilitation

Mari-Anne Wallius^1^, Tea Lallukka^2^, Taina Leinonen^1^, Jouko Remes^1^, Jenni Ervasti^1^

^1^Finnish Institute of Occupational Health, Helsinki, Finland

^2^Department of Public Health, University of Helsinki, Helsinki, Finland

Corresponding author: Mari-Anne Wallius, mari-anne.wallius@ttl.fi

**Supplementary Table 1: Association between level of perceived work ability and risk of full disability pension (2008-2018) among those receiving partial disability pension (N=159, N of events=61). Hazard ratios (HR) and their 95% confidence intervals (CI).** **Cox regression analyses used work ability score as a continuous variable.**

|  | Perceived work ability level |  |
| --- | --- | --- |
|  | HR | 95% CI |
| Model 1^a^ | 0.71 | 0.61–0.83 |
| Model 2^b^ | 0.72 | 0.61–0.84 |
| Model 3^c^ | 0.72 | 0.62–0.84 |
| Model 4^d^ | 0.73 | 0.63–0.85 |

Perceived work ability measured with Work Ability Score (WAS): 0=completely unable to work and 10=work ability at its best.

^a^ Level of work ability, adjusted for baseline age and gender.

^b^ Level of work ability, adjusted for sociodemographic factors (baseline age, gender, occupational class, and marital status).

^c^ Level of work ability, adjusted for health behaviors (baseline smoking, alcohol consumption and leisure-time physical activity).

^d^ Level of work ability, adjusted for baseline age, gender, occupational class, marital status, smoking, alcohol consumption, leisure-time physical activity, and body mass index (BMI).

Leisure-time physical activity, BMI, marital status, occupational class and smoking with imputed values.

Alcohol consumption and leisure-time physical activity were logarithmically transformed.
