# Supplementary material for: Level of and Changes in Perceived Work Ability Among Partial Disability Pensioners and the Risk of Full Disability Pension—A Register-Linked Cohort Study
Source: J Occup Rehabil. 2023 Dec 28;34(3):707–15. doi: 10.1007/s10926-023-10161-z (PMC11364573; doi:10.1007/s10926-023-10161-z)
Supplement: Supplementary file 2 — Supplementary material 2 (DOCX 17.2 kb) [file 10926_2023_10161_MOESM2_ESM.docx]

Level of and changes in perceived work ability among partial disability pensioners and the risk of full disability pension––A register-linked cohort study

Journal of Occupational Rehabilitation

Mari-Anne Wallius^1^, Tea Lallukka^2^, Taina Leinonen^1^, Jouko Remes^1^, Jenni Ervasti^1^

^1^Finnish Institute of Occupational Health, Helsinki, Finland

^2^Department of Public Health, University of Helsinki, Helsinki, Finland

Corresponding author: Mari-Anne Wallius, mari-anne.wallius@ttl.fi

**Supplementary Table 2: Description of study variables by change in perceived work ability groups (between 2008 and 2012)**

|  |  | | Change in work ability | |
| --- | --- | --- | --- | --- |
|  | | All  N* (%) | Unchanged or improved  work ability  N* (%) | Decreased  work ability  N* (%) |
| N | | 80 | 51 | 29 |
| Women (%) | | 73 (91) | 47 (92) | 26 (90) |
| Age (mean) | | 53.7 (SD 4.2) | 53.4 (SD 4.5) | 54.2 (SD 3.5) |
| Occupational class (%) | |  |  |  |
| High | | 35 (44) | 23 (46) | 12 (41) |
| Intermediate | | 29 (37) | 15 (30) | 14 (48) |
| Low | | 15 (19) | 12 (24) | 3 (10) |
| Marital status (%) | |  |  |  |
| Married/cohabiting | | 58 (74) | 40 (78) | 18 (65) |
| Other | | 20 (26) | 11 (22) | 9 (35) |
| Smoking (%) | |  |  |  |
| Non-smoker | | 63 (81) | 41 (82) | 22 (79) |
| Smoker (current) | | 16 (19) | 10 (18) | 6 (21) |
| Alcohol consumption^a, #^ (mean) | | 68.4 (SD 94.3) | 68.9 (98.7) | 67.4 (87.4) |
| Body mass index^b^ (mean) | | 27.1 (SD 4.6) | 26.9 (4.2) | 27.5 (5.2) |
| Physical activity^c, #^ (mean) | | 3.0 (SD 2.2) | 2.8 (1.9) | 3.4 (2.7) |
| Perceived work ability (mean) | | 5.4 (SD 1.7) | 5.0 (SD 1.7) | 6.2 (SD 1.4) |

Work ability measured with Work Ability Score (WAS). Changes in work ability were calculated as the four-year change in WAS by subtracting the 2008 score from the 2012 score, and then categorizing the differences into two classes.

* Numbers vary because some survey responses were missing.

# Non-logarithmic transformed data presented.

^a^ Alcohol consumption calculated as grams per week.

^b^ Body mass index (BMI) calculated from self-reported weight and height.

^c^ Leisure-time physical activity, metabolic equivalent (MET) hours a day.
